# 25 years of surgery in patients with congenital lung malformations: the Rotterdam experience

**DOI:** 10.1007/s00383-026-06503-6

**Published:** 2026-06-15

**Authors:** Marius J. P. Zuidweg, Louis W. J. Dossche, René M. H. Wijnen, J. Marco Schnater

**Affiliations:** Department of Pediatric Surgery, Erasmus MC Sophia Children’s Hospital, Rotterdam, The Netherlands

**Keywords:** Congenital lung malformation, Surgical treatment, Management, Follow-up

## Abstract

**Objective:**

This single-center study aimed to evaluate surgical management of congenital lung malformations (CLM) and its associated perioperative results and short- and long-term outcomes.

**Methods:**

We analyzed data from patients who underwent surgical treatment for CLM. Data were collected within the framework of a structured, prospective longitudinal follow-up program. Length of hospital stay, age at surgery and drain days were analyzed as perioperative outcomes. Surgical approaches and complications were also described.

**Results:**

We included 80 surgically managed patients with CLM over a 25-year period. Eighty-two percent of patients who underwent resection were symptomatic, with respiratory insufficiency (45%) being the primary reason for intervention. Other symptoms were: reduced exercise tolerance, dyspnea and persistent coughing. Reoperation was needed in 2.5% of cases. Median age at intervention was 108 days (IQR 21–463 ) Surgical approaches included thoracotomy (60%), thoracoscopy (29%), embolization (4%) and other interventions (7%). Median length of postoperative hospitalization was 6 days. During their most recent follow-up visit, 21% of the cohort experienced pulmonary symptoms.

**Conclusions:**

A minority of patients with CLM underwent surgical treatment, most of whom were symptomatic. Surgery had low rates of severe complications and a short postoperative stay. Our findings underscore the importance of a long-term management approach.

## Introduction

 Congenital lung malformations (CLM) constitute a diverse group of anomalies arising during fetal development, leading to structural abnormalities in the airways, including congenital pulmonary airway malformation (CPAM), bronchopulmonary sequestration (BPS), bronchogenic cyst (BC), congenital lobar overinflation (CLO), and bronchial atresia (BA) [1]. Hybrid lesions, formed by combinations of aforementioned defects, also occur. Other differential diagnoses that warrant consideration while identifying CLM are pulmonary arteriovenous malformations.

The incidence of CLM is approximately 1 in 2400–2500 births [2]. The number of prenatally diagnosed CLM is rising, most likely due to the introduction of the standard prenatal anomaly scan and the improved ultrasound resolution (since 2007 in The Netherlands) [2]. One in five prenatally diagnosed CLM are symptomatic at birth, showing signs of for example respiratory insufficiency or cardiac overload [3]. In other words; although the majority of children with CLM are asymptomatic at birth, symptoms may develop later in life. In patients with symptomatic CLM, surgical management is widely accepted as standard treatment. Concerning the management of asymptomatic patients, the optimal approach is still disputed: either prophylactic surgery or watchful waiting. In order to answer this question, the CONNECT trial, that is currently being conducted, will compare conservative and surgical management of asymptomatic CPAM patients [4].

Existing literature emphasizes the current practice of tailoring interventions based on institutional expertise and patient-specific factors [5, 6]. Our hospital keeps track of all its patients who underwent major interventions (surgery or ECMO) or were born with congenital malformations, including CLM, through a long-term follow-up program that has been introduced in 1999 [7].

The primary objective of this study was to describe the surgical indications, type of surgical procedures, and postoperative course in selected cases undergoing surgery for CLM at our tertiary referral hospital over the last 25 years. To this end, we identified and described the surgical approaches, as well as postoperative outcomes, including complications and recovery parameters. We also aimed to explore short- and long-term respiratory morbidity outcomes over time.

## Materials and methods

### Patients

A retrospective analysis of prospectively collected data was conducted at the Department of Pediatric Surgery. The cohort comprised all patients who were surgically treated for CLM in our center in the last 25 years.

### Ethical aspects

This study design was approved by the Medical Ethical Review Board of the Erasmus University Medical Center (MEC-2024-0436).

### Study outcomes

All demographic, clinical, radiological, surgical, and follow-up data were extracted from the electronic patient record system. Characteristics of interest were type of CLM, location of the CLM, age at diagnosis, diagnostic modality, symptoms, type of intervention, and (post)operative complications.

Postoperative complications were classified according to the Clavien-Madadi classification (see Appendix A). This classification divides surgical complications into 5 grades; 1 being the least and 5 being the most severe [8]. This score was retrospectively assigned based on data extracted from the electronic medical records.

CLM were divided between CPAM, BPS, CLO, BC and ‘other’. Other entails lung agenesis, bronchial atresia and hybrid lesions.

Symptomatic patients were described as those presenting with one or more of the following: recurrent respiratory infections, mediastinal shift, cardiac complications, growth restriction, or respiratory insufficiency (was defined as: dyspnea regardless of etiology, except infections); including requirement of any respiratory support. Prenatal complications/symptoms and corresponding prenatal interventions were also reported.

### Study design and definitions

This study aimed at describing the evolution over time of perioperative outcomes but also surgical indications, type of surgical procedure, complications, and follow-up in selected patients undergoing surgery. Besides, we sought to determine whether variations in outcomes could be detected before and after the introduction of the routine 20-week prenatal ultrasound screening in 2007.

Our primary outcome is defined as postoperative length of hospital stay (LOS POSTOP). Length of stay at intensive care unit (LOS ICU), age at surgery (AAS), chest drain duration (drain days) are the secondary outcomes. All other variables will be interpreted as patient and (peri)operative characteristics: sex, prematurity, gestational age, duration of follow-up, type CLM, moment of diagnosis, symptomaticity, type of symptoms, onset of symptoms, type of intervention, complications.

Detailed prenatal severity metrics, including CVR, were not included as formal study variables because they were not consistently documented in the pediatric surgical record. In our institution, these data are primarily recorded in maternal obstetric files, which were not covered by the ethical approval for the present pediatric surgical cohort study. Therefore, CVR could not be reliably extracted for the full cohort.

### Description of procedures

The long-term follow-up at the Sophia Children’s Hospital is structured at fixed intervals: outpatient visits at the ages of 6 months, 12 months, 30 months, 5 years, 8 years, 12 years, and 17 years, with continued follow-up in the adult pulmonology outpatient clinic thereafter [7]. During these follow-up visits, the lesion is regularly monitored through X-Thorax and spirometry (for 8 years onwards). According to our current local protocol, an X-ray is performed within 24 h after birth. For asymptomatic patients this is followed by a CTA (Computed Tomography Angiography) after 6 months. In symptomatic patients the CTA is performed earlier (preoperative).

Decisions to pursue either surgical or conservative management were made in multidisciplinary consultation. Antenatal criteria that were used to assess the chance of (immediate) postnatal symptom development were: CVR > 1.6, mediastinal shift, polyhydramnios and concurrent congenital malformations.

To be included in the study, patients had to meet all of the following criteria:

- A confirmed diagnosis of CLM, based on chest computed tomography (CT) imaging and/or histopathological examination.
- Surgical resection or another form of intervention (e.g., embolization) for CLM.
- Availability of preoperative and postoperative medical records.

Patients were excluded if:

- Medical records were unavailable.
- The initial surgical intervention and follow-up was performed outside our center (“Other primary caretaker” in flowchart).
- Lack of informed consent.

Although conservatively managed patients were identified within the broader CLM follow-up population, detailed clinical data on this group were not collected for the present study; therefore, the present analysis was restricted to surgically treated patients (both primary and secondary). Pulmonary arteriovenous malformations were not included in the cohort, as these lesions are generally classified as vascular malformations rather than CLM and are not followed within the institutional structural CLM long-term follow-up program.

### Statistical analysis

Statistical analyses were performed using IBM SPSS Statistics. Categorical variables are presented as frequencies and percentages, and were compared using the Chi-square or Fisher’s exact test, as appropriate. Continuous variables are expressed as mean (with standard deviation) for normally distributed data or median (with interquartile range) for skewed distributions. Normality was determined through a Shapiro-Wilk test.

Subtype-level analyses (all CLM types) were performed secondarily and considered exploratory, with cautious interpretation. These overall differences across all CLM types were evaluated using the Kruskal–Wallis test. Significant results were subjected to Mann–Whitney U tests with Bonferroni correction for multiple comparisons.

A two-sided p-value of < 0.05 was considered statistically significant for the overall analyses.

## Results

**Fig. 1:**
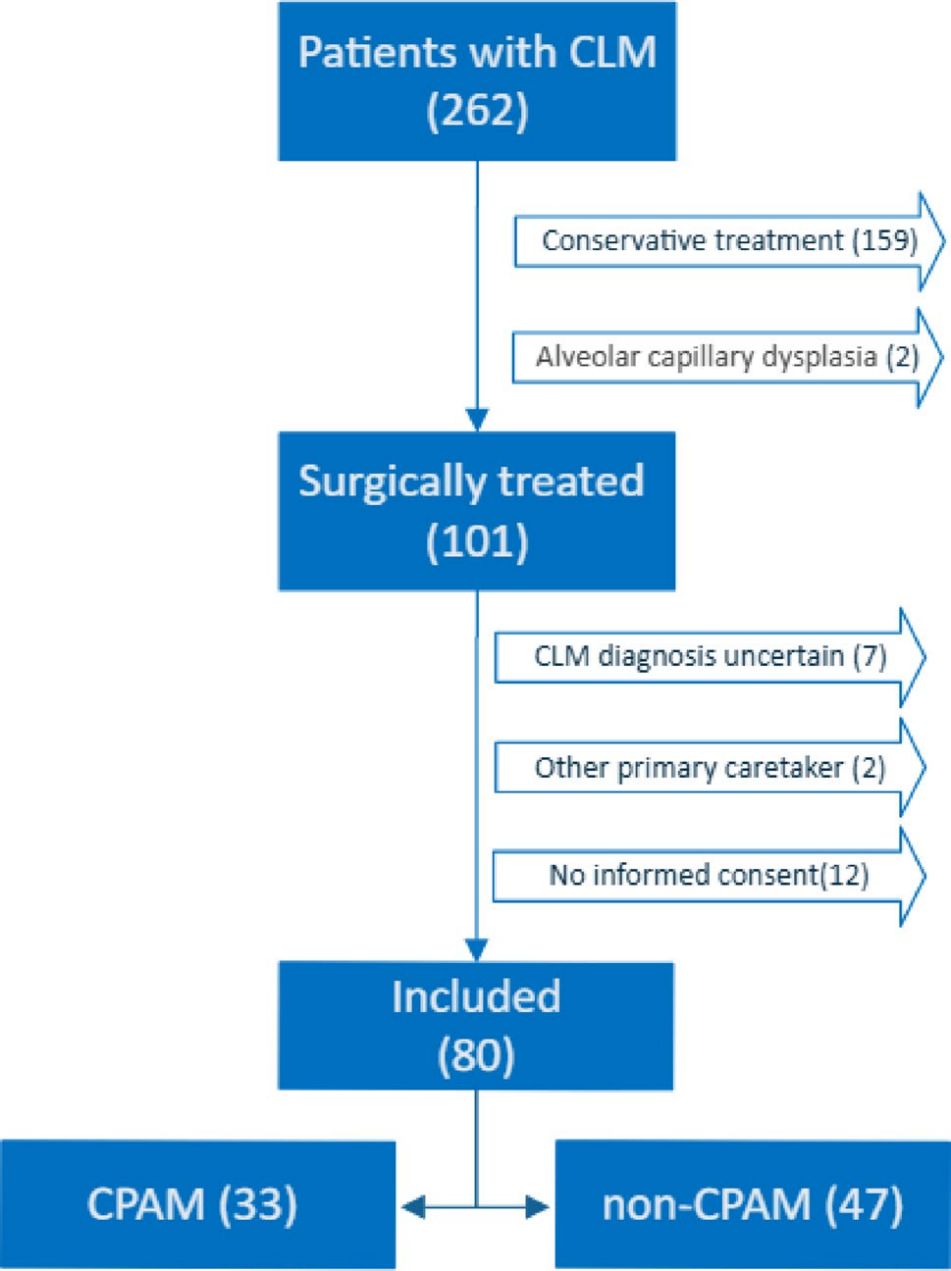
Flowchart of the study

The long-term follow-up program at our center includes 262 patients with CLM, of whom 101 underwent surgical treatment. Of these, 80 met the inclusion criteria for the present study. Seven cases were excluded subsequent diagnostic evaluation did not confirm a CLM diagnosis, for example when the final diagnosis represented a non-congenital thoracic lesion. Two were treated at another hospital initially. Twelve patients did not give informed consent for this study (Fig. 1). Of these, 43% were diagnosed with CPAM and 57% with other types of CLM .

In total, 82 operations were performed. Most patients (83%) were symptomatic at the time of surgery, with respiratory insufficiency (45%) and recurrent respiratory infections (29%) as the most common indications. The proportion of symptomatic patients at the time of surgery increased from 68% before 2007 to 87% after 2007 (*p* = 0.012).

In the asymptomatic group, the rationale behind the operation (when provided), was ‘growth of lesion’. This group consisted out of 5 sequestrations, 2 CPAMs and 1 BC.

In 74% of cases, the diagnosis was made prenatally. This percentage increased from 53% before 2007 to 80% after 2007 (*p* = 0.029). In all surgically treated patients, the diagnosis was confirmed through histopathological examination. In patients with available paired prenatal and postnatal diagnostic data, prenatal and postnatal diagnoses were concordant in the majority of cases (77%). Discordant classifications most frequently involved prenatally suspected CPAM. Among these, seven cases represented major diagnostic shifts, in which prenatally suspected CPAM was ultimately classified as a different CLM subtype: most commonly BPS, and less frequently as BC or a hybrid lesion. In addition, one prenatally suspected BPS was ultimately identified as BC.

Prenatal records mainly distinguished lesions by sonographic appearance: within the prenatally suspected CPAM group, no lesions were documented as macrocystic, two were described as mixed type, and the remainder as microcystic. In all cases, Stocker classification was only determined postnatally.

Prenatal intervention was required in three patients. One fetus with CPAM type 1 and hydrops fetalis at 29 weeks’ gestation, underwent drainage procedures three times (removing 55–60 mL from the CPAM cyst per procedure). A second fetus with CPAM type 3, polyhydramnios and fetal tachycardia, received amnioreduction (2800 mL) at 28 weeks’ gestation. A third patient with CPAM type 1 and hydrops fetalis was treated with betamethasone.

In one case, a malignancy (lepidic type adenocarcinoma) was found. Besides, in 5 cases within our cohort, a KRAS mutation was found in non-malignant CPAM tissue. Furthermore, in one case from our cohort, bronchiectasis was the underlying cause of infections that motivated the resection.

**Table 1:** Patient characteristics

Sex	
Male	46 (58%)
Female	34 (43%)
Premature	
Yes	24%
Median age at birth (wks)	38 + 3
Median follow-up, years	10.4 (IQR 5.2–13.7)
Type CLM	
CPAM	33 (43%)
Type 1	21 (64%)
Type 2	10 (30%)
Type 3	1 (3%)
Not specified	1 (3%)
BPS	17 (21%)
Intralobar	6 (35%)
Extralobar	10 (59%)
Not specified	1 (6%)
BC	10 (13%)
CLO	10 (13%)
Other	10 (13%)

*CLM* congenital lung malformation, *CPAM* congenital pulmonary airway malformation, *BPS* bronchopulmonary sequestration, *BC* bronchogenic cyst, *CLO* congenital lobar overinflation

‘Other’ includes hybrid lesions, bronchial atresia and lung agenesis

The most prevalent CLM was CPAM, with 41% of the cases, followed by BPS at 21%.

In four patients, CLM was concurrent with CDH (Congenital Diaphragmatic Hernia). All four CDHs were paired with a BPS.

In 69 cases, the exact location (lobe) of the lesions was specified. In most cases, CTA was performed first. However, lobar involvement was primarily determined by perioperative findings and postoperative histology. The most common locations of the CLM were the right lower lobe and the left lower lobe. They each accounted for 26% (*n* = 18) of the CLM in our cohort: more than half of the CLM were situated in the lower lobes. The right upper lobe contained 12% (*n* = 8), the right middle lobe 13% (*n* = 9) and the left upper lobe 10% (*n* = 7). Nine CLM, 13%, were multilobar.

As included in Table 2, 66 of the patients were symptomatic at the moment of surgery. The timing of symptom onset (within the symptomatic group) varied: 29% presented at birth, while 21% developed symptoms within the first 28 days (total of 50% in neonatal period). Seventy-seven percent of all patients were operated within the first year of life.

**Table 2:** Clinical presentations

		< 2007	> 2007	Total
Antenatal diagnosis	Yes	10 (53%)	49 (80%)	59 (74%)
Symptomatic (at intervention)	Yes	13 (68%)	53 (87%)	66 (83%)
	Respiratory insufficiency	7 (54%)	23 (43%)	30 (46%)
	Respiratory infections	4 (31%)	15 (28%)	19 (29%)
	Mediastinal shift	1 (8%)	8 (15%)	9 (14%)
	Cardiac complications	–	4 (8%)	4 (6%)
	Growth restrictions	1 (8%)	1 (2%)	2 (3%)
	Other*	–	2 (4%)	2 (3%)
	Unknown**			6 (8%)
				Total (*n* = 62)
Onset of symptoms	At birth			18 (29%)
	< 28 days			13 (21%)
	< 6 months			12 (19%)
	< 12 months			8 (13%)
	< 2 years			4 (6%)
	< 5 years			6 (10%)
	< 10 years			2 (3%)
	> 10 years			3 (5%)

*Other: retrosternal fistula and unspecified symptoms, **Unknown: insufficient documentation in the medical record to reliably determine symptom status

The majority of interventions were performed via thoracotomy (60%), followed by thoracoscopy (29%). Conversion from thoracoscopy to thoracotomy occurred in 33% of cases. A total of 8 conversions from thoracoscopy to thoracotomy were documented. The reasons for conversion were partially related to anatomical complexity and lesion extent, with two cases involving difficult or unexpected anatomical features. In four cases, inadequate visualization or insufficient operative space necessitated conversion, including poor visibility and limited operative overview. In two cases, conversion occurred due to ventilation issues or patient intolerance to insufflation, making thoracoscopic resection unfeasible.

**Table 3:** Surgical management

Category	Subcategory / approach / procedure	< 2007 *N* (%) *N* = 21	> 2007 *N* (%) *N* = 61	TOTAL *N* (%) *N* = 82
Approach	Thoracotomy	18 (86)	31 (51)	49 (60)
	Thoracoscopy	2 (10)	22 (36)	24 (29)
	Conversion	1 (50)	7 (32)	8 (33)
	Embolization	1 (5)	2 (3)	3 (4)
	Other*	–	6 (10)	6 (7)
Procedure	Segmentectomy	–	1 (2)	1 (1)
	Lobectomy	13 (62)	36 (59)	49 (60)
	Pneumonectomy	2 (10)	1 (2)	3 (4)
	Wedge resection	–	2 (3)	2 (2)
	Sequestrectomy / Cystectomy	5 (24)	14 (23)	19 (23)
	Other	1 (5)	7 (12)	8 (10)
Surgical complications	Yes	4 (19)	7 (11)	11 (13)
Complication grades	Grade 1b	–	1 (14)	1 (8)
	Grade 2	1 (25)	2 (29)	3 (23)
	Grade 3a	2 (50)	2 (29)	3 (23)
	Grade 3b	1 (25)	–	1 (8)
	Grade 4	–	1 (14)	1 (8)
Reintervention	Yes	2 (10)	3 (4)	5 (6)

*Other approaches were sternotomy and subcutaneous excision

Embolization and other approaches were used less frequently (Table 3). One patient who underwent embolization for BPS later required surgical intervention due to sepsis. Further details on the patients who underwent embolization procedures can be found in Appendix B.

Lobectomy was the most commonly performed procedure (60%), particularly in CPAM patients (88%). Other procedures included sequestrectomy or cystectomy (23%), pneumonectomy (4%), wedge resection (2%), and segmentectomy (1%) (Table 2).

Surgical complications occurred in 13% (*n* = 11) of patients. The majority of complications were Clavien-Madadi grade 3 (36%) or grade 2 (23%). Out of these complications, two cases were derived from the asymptomatic group (one grade 2, one grade3a). One grade 4 complication was observed. No procedure-related mortality occurred. Reintervention was required in 5 patients (6%), including two reoperations (one underwent embolization followed by surgical resection, and one required reoperation because the initial resection had not completely removed the lesion) and a tension pneumothorax on three occasions (one experienced a tension pneumothorax twice) requiring additional intervention (Table 3).

Postoperative recovery parameters are presented in Table 4. The median ICU stay was 3 days. The median duration of pleural drainage was 3 days. The median length of postoperative hospital stay was 6 days. When comparing postoperative length of stay across all CLM subtypes, an overall difference was observed (Kruskal–Wallis χ²(5) = 11.558, *p* = 0.041). Exploratory post-hoc pairwise comparisons using Mann–Whitney U tests with Bonferroni correction demonstrated a statistically significant difference only between CPAM and BPS, with a longer postoperative length of stay in the latter (Mann–Whitney U = 34.500, two-sided *p* = 0.001). No other pairwise comparisons remained statistically significant after Bonferroni correction.

**Table 4:** Timeline around operation/intervention

	Median duration (days) X [IQR]
LOS POSTOP	6 [4–10]
LOS ICU	3 [2-6.5]
Age at Surgery	108 [21–463]
Drain days	3 [2–4]

*LOS* length of stay, *ICU* intensive care unit, *POSTOP* post-operative

The median follow-up duration was 10.4 years (IQR 5.2–13.7). At the most recent follow-up, 21% of all included patients reported persistent pulmonary symptoms. These symptoms varied between: reduced exercise tolerance (self-reported), dyspnea and persistent coughing. Dyspnea occurred in five patients in each group. Coughing was observed in two patients with CPAM and three with non-CPAM lesions. Severely reduced exercise tolerance was reported exclusively among CPAM patients (*n* = 3).

## Discussion

This study presents the experience of our tertiary referral center managing CLM for over more than two decades.

We showed that, in our center, only 30% of patients known to be born with CLM required surgical intervention for their condition throughout their time in follow-up. The limited proportion of patients undergoing surgery may reflect a cautious, individualized approach to CLM management, with surgery likely reserved for symptomatic or higher-risk cases. In our surgical cohort, 83% of CLM patients were symptomatic, most frequently presenting with respiratory insufficiency or infections.

We hypothesized that the introduction of routine 20-week prenatal ultrasound screening, in 2007, had caused an increase in the proportion of asymptomatic patients who underwent surgery. However, when comparing patients operated before and after the introduction of routine 20-week anomaly screening, we found the following difference: the proportion of symptomatic patients at the moment of surgery rose from 68% to 87% (*p* = 0.012). This might be contributable to the increasingly conservative approach for asymptomatic lesions over time. As a result of the retrospective design, this era-based comparison is susceptible to changes in detection, referral patterns, and surgical selection thresholds over time, and this interpretation should therefore be considered hypothesis-generating rather than definitive.

In our institution, conservative management has been the most common treatment in asymptomatic children for years. This is in line with other reports supporting observation in selected cases, given the relatively indolent natural history of many lesions [9–11]. Nonetheless, as demonstrated by Kantor et al., up to 25% of prenatally diagnosed asymptomatic (CPAM) lesions can develop symptoms later in childhood, highlighting the need for close follow-up [12]. Our management strategies for asymptomatic CPAM cases, are currently decided based on the protocol of the CONNECT Trial [4].

In our cohort, CPAM was the most frequent CLM type (41%), followed by BPS (21%), CLO (13%), and BC (13%). Looking specifically at the small group of asymptomatic CLM patients, BPS was the most common subtype (63%). Within BPS, we noted a higher proportion of extralobar (60%) over intralobar (35%) sequestrations (one case was unspecified). This distribution of sequestrations differs from the prevalence that is typically reported in the literature. In most series, intralobar sequestrations (ILS) are more common, accounting for around 80–90% of cases, whereas extralobar lesions (ELS) make up only 10–20% [13, 14]. ILS has a higher risk of infection, due to retained communication with the airways, eventually through pores of Kohn, while ELS rarely infect but are often resected because they are technically easier to remove. The predominance of ELS can partially be explained by the fact that 4 cases of ELS were patients with CDH. When disregarding these specific cases, the ILS: ELS ratio in our surgical cohort would be 1:1. This distribution is consistent with findings from a European survey among healthcare professionals, which reported that intralobar and extralobar sequestrations are generally regarded as clinically equivalent in their management [15]. The presence of hybrid lesions and cases associated with CDH further illustrate the complexity of CLM management.

In our cohort, we identified KRAS mutations in non-malignant CPAM tissue in 5 cases, highlighting the potential for molecular alterations in benign lesions. Additionally, two patients were diagnosed with malignant transformation (lepidic type adenocarcinoma), highlighting the rare potential for malignancy in CLM. These findings indicate the need for future research to better stratify the risks of malignancy and other long-term complications in CLM patients. Given the possibility of malignant transformation and other pulmonary outcomes, long-term follow-up is essential for all patients, regardless of the lesion’s initial presentation or histological features.

Overall, thoracotomy remained the most common surgical approach (60%), though thoracoscopic usage increased after 2007 (10% before 2007 and 36% after 2007). While our center has embraced thoracoscopy over time, open thoracotomy retains a role; probably especially in complex or early-period cases.

Notably, conversion to open surgery was necessary in one third of the thoracoscopic cases, which is higher than reported in another series [16]. In our cohort, conversions were primarily related to (1) anatomic complexity or a larger/more extensive lesion than anticipated, (2) limited visualization/insufficient overview, and (3) ventilation intolerance during insufflation. Future (multicenter) studies could systematically assess predictors of failed thoracoscopy by integrating preoperative imaging features, lesion extent, and intraoperative exposure/ventilation tolerance in order to support pragmatic selection criteria and counseling.

Furthermore, our complication rate of approximately 13% is within the range reported in other series, and severe (grade 3 or higher) complications were infrequent (6%) [16].

A key concern in the management of CLM is the long-term impact of surgery. When comparing lesions types, long-term outcomes after resection, including pulmonary function and quality of life, have generally been shown to be comparable [17, 18]. Rates of lower respiratory tract infections are comparable between surgically and conservatively treated patients [19]. In our cohort, 21% of all patients still showed various pulmonary symptoms, which shows the importance of closely monitoring this group of patients, also after resection.

Since our cohort focused on surgically treated cases, the long-term outcomes of conservatively managed asymptomatic CLM patients remain uncertain and should be studied further. The present findings should therefore be interpreted as reflecting outcomes within a selected operated cohort, rather than as evidence regarding the relative merits of operative versus non-operative management in the broader CLM population. Comparison with conservatively managed patients would provide valuable additional insight, particularly in interpreting changes in management strategies over time and persistent pulmonary symptoms at long-term follow-up. Literature suggests that asymptomatic patients managed conservatively may face risks such as recurrent infections, growth retardation, and, rarely, malignant transformation [1]. Given these potential risks, long-term follow-up in all CLM patients, regardless of initial symptom presentation, is crucial to monitor for these complications and to guide management decisions.

This study has several important limitations that should be considered when interpreting the results.

Our cohort represents solely surgically treated patients and is not generalizable to all prenatally detected CLM. This selection likely enriches for symptomatic patients and lesions perceived to be higher risk or more complex, which may bias observed characteristics and outcomes.

Taking into account the 25-year inclusion period, changes in prenatal detection, perioperative care, and surgical technique are likely to have influenced the composition of the operated cohort and the management approaches used over time. We therefore used the pre-/post-2007 stratification, also as a pragmatic way to assess consistency of key cohort characteristics and outcomes across two eras within our institution. These era-based findings are interpreted descriptively, acknowledging that temporal changes in referral pathways, documentation, and practice patterns may confound direct comparisons.

Besides, in 7% of the cases, not all relevant information was available from the follow-up data. Nevertheless, the long duration of observation and the inclusion of a wide spectrum of lesions provide valuable insight into real-world practice.

In conclusion, our findings reflect a selective surgical strategy, with operative management reserved for a minority of CLM patients. In this 25-year single-center cohort of surgically treated congenital lung malformations, surgery was predominantly performed in symptomatic or higher-risk patients, with lobectomy being the most common procedure and thoracotomy the most frequent approach. Postoperative outcomes were generally favorable, with a median postoperative hospital stay of 6 days and a low rate of severe complications. Long-term follow-up remains essential, as a proportion of patients continue to experience respiratory symptoms post-resection. Further prospective studies are needed to better define the optimal timing and modality of treatment, to understand the long-term consequences for symptom development and morbidity and for stratifying patients into high- and low-risk groups that might require a different approach/treatment.

## Data Availability

The data presented in this study are available on request from the corresponding author.

## Appendix A

### Clavien–Madadi classification

**Table:**

Grade	Subtype	Definition
I	A	Any deviation from the planned course due to management and/or organizational problems.
I	B	Any deviation from the planned clinical course with the need for pharmacological treatment, such as antiemetics, antipyretics, analgesics, diuretics, electrolytes, and physiotherapy. This grade also includes wound infections opened at the bedside.
II		Requiring pharmacological treatment with drugs other than those listed for Grade Ib. Blood transfusions and parenteral nutrition are also included.
III	A	Endoscopic and radiological-guided interventions. Interventions via laparoscopy/thoracoscopy.
III	B	Interventions under anesthesia (other than those listed for Grade IIb). Interventions via laparotomy/thoracotomy.
IV		Multi-organ dysfunction*
V		Death of a patient.

*Multi-organ dysfunction is defined as the concurrent dysfunction of two or more organs or systems including respiratory, cardiovascular, hematological, neurological, gastrointestinal, hepatic, and renal

### Appendix B

**Table:**

Type CLM	Indication for embolization	Age at embolization (days)	Embolization definitive treatment?
BPS	Congenital heart disease (Multiple congenital anomalies)	34	No
BPS	Left vertricular volume overload	1252	Yes
BPS (multiple)	Pulmonary Hypertension (Multiple congenital anomalies)	72	Yes (for this problem)
